# Giant Cell Tumor of the Tenosynovium: An Uncommon Presentation in the Forefoot

**DOI:** 10.1155/cro/9966160

**Published:** 2026-05-15

**Authors:** Prasamsha Sitaula, Ojas Thapa, Sauharda Sitaula, Sarvesh Dahal

**Affiliations:** ^1^ Department of Orthopaedics, Nepal Medical College and Teaching Hospital, Kathmandu, Nepal; ^2^ Department of Orthopaedics, Nepalese Army Institute of Health Sciences, Kathmandu, Nepal, naihs.edu.np; ^3^ Department of Orthopaedics, Nobel Medical College and Teaching Hospital, Biratnagar, Nepal, nobelmedicalcollege.com.np

## Abstract

Tenosynovial giant cell tumor (TSGCT) is a benign, slow‐growing solid soft tissue tumor that arises from the tenosynovium of a tendon sheath or the synovium of a diarthrodial joint. TSGCT accounts for 1.6% of all soft tissue tumors and typically arises in the limbs, with the most common location being the hand, whereas occurrence in the foot is rare. Local complete resection of the lesion is an effective treatment for TSGCT. However, if left untreated, the tumor can lead to persistent pain, limited movement, joint destruction, and osteoarthritis. We present the case of a young gentleman diagnosed with a TSGCT of the forefoot, a rare but locally aggressive lesion that was successfully treated with complete excision.

## 1. Introduction

Tenosynovial giant cell tumor (TSGCT) is a benign, slow‐growing solid soft tissue tumor that arises from the tenosynovium of a tendon sheath or the synovium of a diarthrodial joint [1, 2]. Most of these tumors are benign but can be locally aggressive [3]. They typically occur in patients between the ages of 20 and 40, with a slight predominance in females [4]. The classification is determined by location (intra‐articular or extra‐articular), the specific area affected, and the growth pattern, which can be either localized or diffuse [5]. TSGCT accounts for 1.6% of all soft tissue tumors and typically arises in the limbs, with the most common location being the hand, whereas occurrence in the foot is rare [6]. Patients typically exhibit a painless, solitary swelling. X‐rays are essential for diagnosis, as standard radiographs can identify bone abnormalities. Once the diagnosis is suspected based on clinical findings and X‐ray results, magnetic resonance imaging (MRI) is considered the most valuable imaging technique for diagnosis and preoperative assessment.

Most commonly, it presents as a single nodular, yellow–brown outgrowth of the synovial membrane [7]. Histopathologically, the lesions are characterized by polyhedral stromal cells, multinucleated giant cells, round oval mononuclear cells, and hemosiderin deposits [8]. Local complete resection of the lesion is an effective treatment for TSGCT [9]. However, if left untreated, the tumor can lead to persistent pain, limited movement, joint destruction, and osteoarthritis [10]. The recurrence rate after treatment may range from 14% to 55% [9], which may be due to inadequate excision, bony erosion involvement, lesion size exceeding 2 cm, and tendon or neurovascular involvement [11–13].

We present the case of a young gentleman diagnosed with a TSGCT of the forefoot, a rare but locally aggressive lesion that was successfully treated with complete excision.

## 2. Case Report

**Figure 1:**
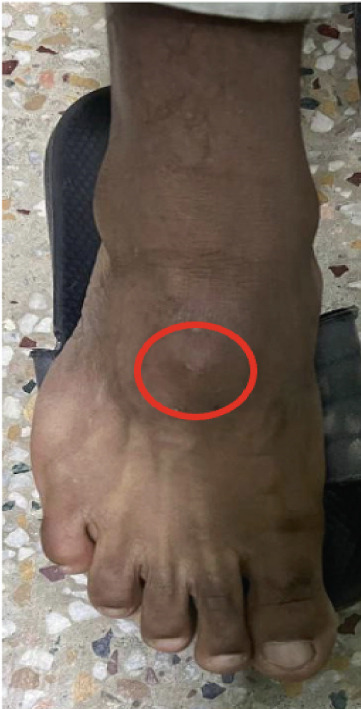
Swelling over the dorsal surface of forefoot.

**Figure 2:**
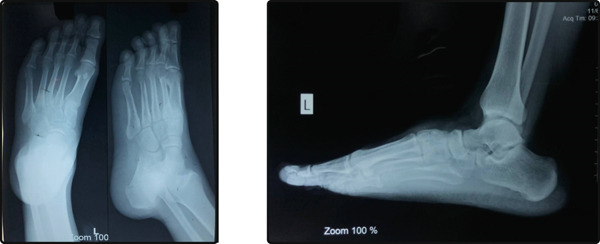
No evidence of bony erosion.

We present the case of a 23‐year‐old male who had been experiencing swelling on the dorsum of his left foot for 2 years. The swelling gradually increased in size, causing discomfort when wearing socks or shoes. The patient had no history of trauma, fever, or any predisposing factors. On physical examination, a well‐defined, oval swelling is visible over the dorsum of the left foot at the level of the third metatarsal base. The overlying skin appears smooth with no erythema or ulceration. No visible deformity of adjacent toes is noted (Figure 1). The skin over the mass was smooth, with no signs of inflammation. The lesion was nonpulsatile, and there were no sensory or motor deficits. Radiological imaging showed no obvious bony erosion, periosteal reaction, or calcification. Cortical margins were preserved, and there was no obvious bony abnormality, and laboratory tests were within normal limits (Figure 2). MRI is considered the gold standard for preoperative mapping, particularly in the foot, due to its ability to delineate soft tissue extent, tendon sheath involvement, and hemosiderin deposition. In this case, MRI was not performed due to strong clinical suspicion of a benign, localized lesion and absence of concerning radiographic features. This represents a limitation of our study. The decision to proceed with excisional biopsy was considered safe given the superficial location, well‐circumscribed nature, lack of neurovascular involvement, and benign radiographic appearance. Plain radiographs (anteroposterior and oblique views) of the foot showed no obvious bony erosion, periosteal reaction, or calcification. Given the strong clinical suspicion of a benign tumor and the absence of indications to assess deeper structures or exclude aggressive behavior, a confirmatory excisional biopsy was performed to establish a definitive diagnosis and provide treatment.

**Figure 3:**
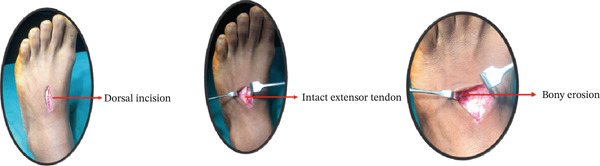
Intraoperative pictures showing the dorsal incision, intact extensor tendon and underlying bony erosion.

Under spinal anesthesia, a tourniquet was applied, and after taking full aseptic precautions, an incision was made over the mass. A 4‐cm longitudinal dorsal incision was made over the lesion. A pneumatic tourniquet was applied at 250 mmHg for approximately 60 min. Careful dissection was performed to protect the extensor tendons and surrounding neurovascular structures. The well‐circumscribed lesion was excised en bloc, followed by a partial synovectomy with the extensor tendon found to be intact, though bony erosion was observed. Meticulous hemostasis was achieved using bipolar cautery. Histopathological examination confirmed negative margins, with the closest margin measuring 0.2 mm. Although iron staining for hemosiderin and immunohistochemistry could have provided additional support, they were not performed as the diagnosis was definitive based on characteristic histomorphological features (Figure 3).

**Figure 4:**
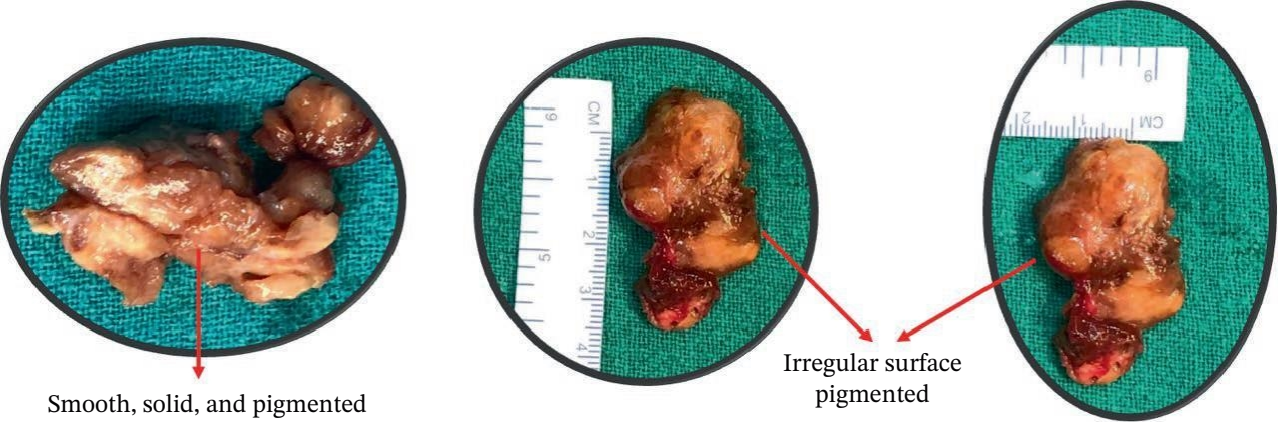
Gross appearance.

The excised mass was sent for histopathological examination. Grossly, the tissue measured 4 × 3.5 × 0.8 cm. The outer surface was irregular, with a dark brown to gray–white appearance. The cut section revealed a smooth, solid, homogeneous dark–brown area (Figure 4).

**Figure 5:**
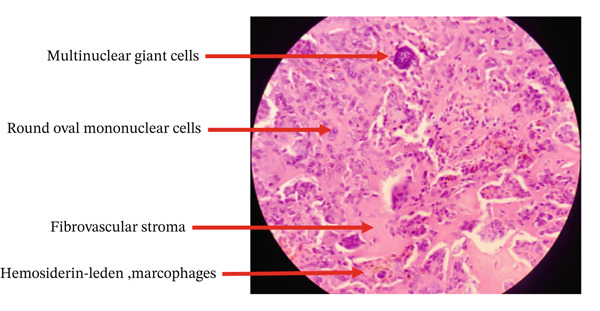
Microscopic finding.

Microscopic examination showed closely packed, medium‐sized polyhedral cells, along with a variable admixture of giant cells containing fat and hemosiderin. These atypical cells exhibited moderate pleomorphism, with an increased nucleus‐to‐cytoplasm ratio, enlarged round‐to‐oval nuclei, vesicular chromatin, and prominent nucleoli. The cytoplasm was moderate in amount. Numerous multinucleated foreign‐body type giant cells were observed, alongside a few pigment‐laden macrophages. Areas of sclerosis with foci of osteoid formation were also noted. No evidence of malignancy was found (Figure 5).

The findings were suggestive of a TSGCT as at low magnification (×40), a well‐circumscribed lesion composed of cellular proliferation is seen, which on higher magnification (×100–×400) demonstrates multinucleated giant cells, polyhedral stromal cells, and hemosiderin‐laden macrophages.

A comparative analysis of TGCT against fibroma of the tendon sheath, ganglion cyst, lipoma, and synovial sarcoma is presented in Table 1, highlighting key diagnostic differences in clinical presentation, imaging (USG/MRI), and histopathological/IHC profiles.

**Table 1:** Differential diagnosis of tenosynovial giant cell tumor (TGCT).

Feature	TGCT	Fibroma of tendon sheath	Ganglion cyst	Lipoma	Synovial sarcoma
Nature	Benign, locally aggressive	Benign fibrous	Cystic	Benign adipose	Malignant
Clinical	Firm, slow growing	Firm, attached	Fluctuant	Soft	Painful, enlarging
USG	Solid, hypoechoic	Solid	Anechoic	Echogenic	Heterogeneous
MRI	Low signal (hemosiderin)	Low signal	Fluid signal	Fat signal	Heterogeneous, invasive
Histology	Giant cells, hemosiderin	Dense collagen	No cells	Adipocytes	Atypical spindle cells
IHC	CD68+, hemosiderin	Variable	Not needed	S100+	TLE1+, cytokeratin+

This case report has been prepared in accordance with the CARE guidelines.

## 3. Discussion

A TGCT is a benign tumor that occurs in the joints, tendons, and synovial bursas. The most common sign is an asymptomatic mass, although additional symptoms like pain and restricted joint motion can occur [1].

The differential diagnosis should consider other tumors such as lipoma, ganglia, or fibromas. Imaging plays a crucial role in differentiating TSGCT from other soft tissue tumors in the foot [1]. An X‐ray is essential, as bone abnormalities are present in 33% of cases and can be detected [12]. Ultrasonography helps differentiate solid from cystic masses, revealing TSGCT as a homogeneous, solid, and hypoechoic mass [14]. An MRI must be conducted after suspecting a diagnosis based on clinical data and X‐ray findings. Unfortunately, in our cases, an MRI was overlooked in favor of an excisional biopsy based solely on clinical and X‐ray findings. MRI, however, is crucial for diagnosis and preoperative planning, revealing tumor characteristics like iso‐to‐hypointense signals on T1W images and variable signals on T2W images due to hemosiderin pigments [15].

Macroscopically, the tumor appears as a gray–white, encapsulated, lobulated mass with spots of yellow and brown, varying according to the amount of hemosiderin‐laden histiocytes [4]. In our case, the mass was smooth and solid, with a homogeneous dark–brown area. Microscopically, it consists of hemosiderin‐containing multinucleated giant cells, histiocyte‐like and fibroblast‐like cells, xanthoma cells, and vascular structures [4], similar findings were observed in our case with closely packed polyhedral cells, giant cells containing fat, and hemosiderin.

Complete local resection is effective in treating TSGCT and helps minimize the risk of local recurrence [9]. Despite its effectiveness, the recurrence rate is between 4% and 44% [16].

Overall, the recurrence rate of TGCT is generally higher in the ankle and subtalar joints, likely due to the tumor′s behavior and the challenges associated with achieving complete surgical excision in these areas [17]. According to Bruns et al., the foot demonstrated the lowest recurrence rate among large joints: 4% (7 out of 173 cases), followed by a higher rate of 9% (16 out of 173 cases) in the ankle and subtalar joints [18].

In cases of recurrence, re‐excision is recommended, since conversion to malignancy has not been reported even after multiple recurrences [16]. Palmerini et al. emphasized that complete resection is the primary treatment, but they also advised maintaining a balance between ensuring adequate surgical margins and preserving function [11].

In our case, no recurrence was observed during the 2‐year follow‐up period, likely due to complete surgical excision of the tumor. Meticulous dissection and removal of the lesion in its entirety, along with clear margins and excision of the involved synovial tissue, minimized the risk of residual disease. Regular postoperative clinical evaluations and radiological X‐ray monitoring consistently confirmed the absence of any signs of recurrence throughout the follow‐up period.

## 4. Conclusion

When evaluating a soft tissue tumor around the foot, a TSGCT must be included in the differential diagnosis. MRI is the preferred method for diagnosing and preoperatively assessing these tumors. However, a definitive diagnosis requires histological examination, and complete resection is necessary to prevent recurrence.

## Funding

No funding was received for this manuscript.

## Ethics Statement

As this is a single‐patient case report, formal Institutional Review Board (IRB) approval was not required. Written informed consent was obtained from the patient for publication of clinical details and accompanying images.

## Conflicts of Interest

The authors declare no conflicts of interest.

## Data Availability

The data that support the findings of this study are available from the corresponding author upon reasonable request.

## References

[1] Goni V. , Gopinathan N. R. , Radotra B. D. , Viswanathan V. K. , and Logithasan R. K. , Giant Cell Tumour of Peroneus Brevis Tendon Sheath - a Case Report and Review of Literature, BMJ Case Reports. (2012) 2012, bcr0120125703, 10.1136/bcr.01.2012.5703, 2-s2.0-84865342888, 22802558. PMC3417015 22802558

[2] Chou L. B. , Ho Y. Y. , and Malawer M. M. , Tumors of the Foot and Ankle: Experience With 153 Cases, Foot and Ankle International. (2009) 30, no. 9, 836–841, 10.3113/FAI.2009.0836, 2-s2.0-70349118283, 19755066. 19755066

[3] Ravi V. , Wang W. L. , and Lewis V. O. , Treatment of Tenosynovial Giant Cell Tumor and Pigmented Villonodular Synovitis, Current Opinion in Oncology. (2011) 23, no. 4, 361–366, 10.1097/CCO.0b013e328347e1e3, 2-s2.0-79958807634. 21577109

[4] Fletcher C. D. M. , Bridge J. A. , Hogendoorn P. C. W. , and Mertens F. , Pathology and Genetics of Tumours of Soft Tissue and Bone, World Health Organization; International Agency for Research on Cancer, 2013, 4th edition, IARC Press, 100–104.

[5] Mendenhall W. M. , Mendenhall C. M. , Reith J. D. , Scarborough M. T. , Gibbs C. P. , and Mendenhall N. P. , Pigmented Villonodular Synovitis, American Journal of Clinical Oncology. (2006) 29, no. 6, 548–550, 10.1097/01.coc.0000239142.48188.f6, 2-s2.0-33845485066. 17148989

[6] Occhipinti E. , Heinrich S. , and Craver R. , Giant Cell Tumor of Tendon Sheath Arising in the Toe, Fetal and Pediatric Pathology. (2004) 23, no. 2-3, 171–179, 10.1080/15227950490890441, 2-s2.0-15044365928, 15768862. 15768862

[7] Rao A. S. and Vigorita V. J. , Pigmented Villonodular Synovitis (Giant-Cell Tumor of the Tendon Sheath and Synovial Membrane). A Review of Eighty-One Cases, Journal of Bone and Joint Surgery. (1984) 66, no. 1, 76–94, 10.2106/00004623-198466010-00012, 2-s2.0-0021330107, 6317696. 6317696

[8] Flandry F. , Hughston J. , McCann S. , and Kurtz D. , Diagnostic Features of Diffuse Pigmented Villonodular Synovitis of the Knee, Clinical Orthopaedics and Related Research. (1994) 298, 212–220. 8118978

[9] Verspoor F. , Van Der Geest I. , Vegt E. , Veth R. , Van Der Graaf W. , and Schreuder H. , Pigmented Villonodular Synovitis: Current Concepts About Diagnosis and Management, Future Oncology. (2013) 9, no. 10, 1515–1531, 10.2217/fon.13.124, 2-s2.0-84885667746, 24106902. 24106902

[10] Ono Y. , Miyakoshi N. , Tsuchie H. , Nagasawa H. , Nanjo H. , and Shimada Y. , Pigmented Villonodular Synovitis Around the Elbow Joint That Required Upper Arm Amputation, Journal of Medical Cases. (2020) 11, no. 7, 201–203, 10.14740/jmc3503, 34434397. 34434397 PMC8383624

[11] Palmerini E. , Staals E. L. , Maki R. G. , Pengo S. , Cioffi A. , Gambarotti M. , Picci P. , Daolio P. A. , Parafioriti A. , Morris C. , Antonescu C. R. , Gronchi A. , Casali P. G. , Donati D. M. , Ferrari S. , and Stacchiotti S. , Tenosynovial Giant Cell Tumour/Pigmented Villonodular Synovitis: Outcome of 294 Patients Before the Era of Kinase Inhibitors, European Journal of Cancer. (2015) 51, no. 2, 210–217, 10.1016/j.ejca.2014.11.001, 2-s2.0-84925446588, 25465190. 25465190

[12] Gouin F. and Noailles T. , Localized and Diffuse Forms of Tenosynovial Giant Cell Tumor (Formerly Giant Cell Tumor of the Tendon Sheath and Pigmented Villonodular Synovitis), Orthopaedics and Traumatology: Surgery and Research. (2017) 103, no. 1, S91–S97, 10.1016/j.otsr.2016.11.002, 2-s2.0-85008415032, 28057477. 28057477

[13] Fotiadis E. , Papadopoulos A. , Svarnas T. , Akritopoulos P. , Sachinis N. P. , and Chalidis B. E. , Giant Cell Tumour of Tendon Sheath of the Digits. A Systematic Review, A Systematic Review Hand. (2011) 6, no. 3, 244–249, 10.1007/s11552-011-9341-9, 2-s2.0-80051473335. 22942846 PMC3153624

[14] Middleton W. D. , Patel V. , Teefey S. A. , and Boyer M. I. , Giant Cell Tumors of the Tendon Sheath: Analysis of Sonographic Findings, American Journal of Roentgenology. (2004) 183, no. 2, 337–339, 10.2214/ajr.183.2.1830337, 2-s2.0-3242805952, 15269021. 15269021

[15] De Beuckeleer L. , De Schepper A. , De Belder F. , Goethem J. V. , Marques M. C. B. , Broeckx J. , Verstraete K. , and Vermaut F. , Magnetic Resonance Imaging of Localized Giant Cell Tumour of the Tendon Sheath (MRI of Localized GCTTS), European Radiology. (1997) 7, no. 2, 198–201, 10.1007/s003300050134. 9038114

[16] Chen Y. U. , Yu X. C. , Xu S. F. , and Wang B. , Giant Cell Tumor of the Tendon Sheath Originating From the Ankle Capsule: A Case Report and Literature Review, Oncology Letters. (2016) 11, no. 5, 3461–3464, 10.3892/ol.2016.4377, 2-s2.0-84976867196, 27123136. 27123136 PMC4841104

[17] Zhang Y. , Huang J. , Ma X. , Wang X. , Zhang C. , and Chen L. , Giant Cell Tumor of the Tendon Sheath in the Foot and Ankle: Case Series and Review of the Literature, Journal of Foot and Ankle Surgery. (2013) 52, no. 1, 24–27, 10.1053/j.jfas.2012.09.008, 2-s2.0-84871510427, 23085383. 23085383

[18] Bruns J. , Ewerbeck V. , Dominkus M. , Windhager R. , Hassenpflug J. , Windhagen H. , Hovy L. , Loehr J. , Krauspe R. , and Duerr H. R. , Pigmented Villonodular Synovitis and Giant-Cell Tumor of Tendon Sheaths: A Binational Retrospective Study, Archives Of Orthopaedic And Trauma Surgery. (2013) 133, no. 8, 1047–1053, 10.1007/s00402-013-1770-1, 2-s2.0-84880921551, 23681468. 23681468

